# From bench to bedside: unlocking the anti-inflammatory, antioxidant, and anticancer promise of curcumin in gynecology

**DOI:** 10.3389/fmed.2026.1761721

**Published:** 2026-02-20

**Authors:** Nazlı Tunca Sanlier, Koray Gorkem Sacinti, İnci Turkoglu, Nevin Sanlier

**Affiliations:** 1 Department of Obstetrics and Gynecology, Niğde Çiflik State Hospital, Niğde, Türkiye; 2 Department of Obstetrics and Gynecology, Obstetrics and Gynecology, Yale School of Medicine, New Haven, CT, United States; 3 Department of Epidemiology, Hacettepe University Faculty of Medicine, Ankara, Türkiye; 4 Department of Public Health, Hacettepe University Faculty of Medicine, Ankara, Türkiye; 5 Department of Nutrition and Dietetics, Hacettepe University Faculty of Health Sciences, Ankara, Türkiye; 6 Department of Nutrition and Dietetics, School of Health Sciences, Ankara Medipol University, Ankara, Türkiye

**Keywords:** curcumin, endometriosis, gynecological cancer, menopause, polycystic ovary syndrome, polyphenols, premenstrual syndrome

## Abstract

Curcumin, a bioactive polyphenol derived from turmeric (*Curcuma longa*), has garnered substantial attention for its potent anti-inflammatory, antioxidant, and antineoplastic properties. This review explores the therapeutic potential of curcumin in gynecologic health, with a focus on its role in the management of ovarian, cervical, and endometrial cancers, as well as benign conditions such as endometriosis, polycystic ovary syndrome, premenstrual syndrome, and menopausal symptoms. A literature review was conducted on the health effects of gynecology. Relevant articles were identified through systematic searches in major biomedical databases, including PubMed, Scopus, Cochrane Library, Embase, and Web of Science databases for studies reporting the relationship between curcumin and some in gynecological diseasess as of 2025. Curcumin modulates key inflammatory signaling pathways, reduces oxidative stress, and exerts antiproliferative effects, making it a promising adjunct in the treatment of both neoplastic and inflammatory gynecologic disorders. Nevertheless, clinical translation remains limited by challenges such as poor bioavailability and a paucity of large-scale, randomized controlled trials. Emerging evidence supports the integration of curcumin into multimodal treatment strategies, particularly in oncology and chronic inflammatory conditions. In light of the need to improve treatment efficacy and enhance patients’ quality of life, the exploration of novel adjuvant therapeutic strategies is highly warranted. Recent studies have demonstrated the beneficial effects of curcumin and its novel analogues across a range of gynecologic diseases, while advances in formulation technologies have led to improved pharmacokinetic profiles and therapeutic outcomes. Nevertheless, further robust clinical investigations are required to optimize curcumin formulations, enhance bioavailability, and establish evidence-based guidelines for its integration into gynecologic care. This review synthesizes current evidence and highlights the underlying molecular mechanisms responsible for the observed effects, aiming to support the rational development of curcumin-based strategies in gynecology.

## Introduction

Curcumin, the principal bioactive polyphenol extracted from the rhizome of *Curcuma longa* (turmeric), has been the focus of extensive biomedical research due to its wide spectrum of pharmacological properties, including antioxidant, antimicrobial, anti-inflammatory, and antineoplastic effects. Curcumin, the main active ingredient in turmeric, has attracted interest due to its wide range of therapeutic benefits that extend beyond its recognized anticancer properties (1–8). Similar to other plant-derived polyphenols, curcumin exemplifies the broad therapeutic potential of phytochemicals in oncology (7). These properties are of particular relevance in gynecology, where oxidative stress, chronic inflammation, and tumorigenesis underlie numerous pathological conditions. Curcumin exerts its biological effects by modulating key molecular targets such as transcription factors, cytokines, kinases, enzymes, and cell surface receptors thereby demonstrating therapeutic versatility in addressing multifactorial diseases (8–11).

Despite its broad mechanistic potential, curcumin’s clinical translation has been hindered by its inherently low aqueous solubility, rapid metabolism, and poor systemic bioavailabilit (12) (Table 1). These pharmacokinetic limitations significantly reduce its therapeutic efficacy *in vivo*. Accordingly, recent advancements have focused on the emphasized the design of innovative delivery platforms and optimized formulations including nanoparticles, liposomes, and phospholipid complexes to enhance curcumin’s bioavailability and clinical utility. Formulation approaches using nanotechnology has recently attracted interest as a promising approach to improve the pharmacokinetic behavior and site-specific delivery of curcumin through encapsulation in lipid-based systems, polymeric nanoparticles, nanomicelles, and phytosomes (13, 14). Because the majority of the curcumin-based nanoformulation evidence is still in the conceptual stage, there are still numerous issues impeding the provision of nanocurcumin as a possible therapeutic option. Although preclinical studies of these nanoformulation and targeting strategies have shown promising results, further research is needed to validate their clinical applicability and therapeutic reliability.

**TABLE 1:** Some studies highlighting the relationship between curcumin and gynecological diseases.

Study type	Models	Dose and duration	Effects/mechanisms	References
**Ovarian cancer**				
*In vitro*	SKOV3 cells	10, 20, 30, 40, and 50 μM, 6, 12, and 24 h	Inhibiting migration and invasion ↓STAT3, fascin	(148)
*In vivo*	SKOV3 and A2780 cells BALB/c athymic mice with A2780 cells	10, 20 and 40 μM, 24, 48 and 72 h; 15 mg/kg/2 days, 5 weeks	Inhibiting proliferation; promoting apoptosis ↓PCNA, miR-320a, ↑Bax, Cleaved-caspase-3, circ- PLEKHM3, SMG1	(149)
*In vitro*	SKOV3 cells line	SKOV3 cells were treated with DMSO, 10 μM 5-aza-2′-d DAC, 5 μM DAC, 20 μM curcumin, 5 μM DAC with 20 μM curcumin for 96 h	↓Tumor growth ↓Migration ↓Invasion ↓ Wnt/β-catenin	(150)
*In vitro*	ES-2, SKOV3 cell line	ASA/Cur-coloaded mPEG-PLGA nanoparticles 5 μg/mL	↑Apoptosis induction ↑ Antitumor activity	(151)
	SKOV3 and A2780 cell line	curcumin, alone or combined with paclitaxel	↑Apoptosis ↓Inhibiting cell proliferation regulates the miR-9–5p/BRCA1 axis ↓miR-9–5p expression ↑ B RCA1 expression	(152)
**Cervical cancer**				
*In vitro*	Siha cells	5, 15, 30 and 50 μM, 6, 12, 24 and 48 h	↓Proliferation ↑ G2/M cell cycle arrest ↑Apoptosis ↑Autophagy ↓cyclins B1 ↑cdc25 ↑ROS ↑ p62 ↑LC3I/II	(153)
*In vitro*	Siha cells	20 μM, 72 h	↓ EMT and migration ↓N-cadherin ↓Vimentin ↓slug, Zeb1 ↓ PIR ↓Pirin ↑E-cadherin	(154)
Advanced cervical cancer patients (*n* = 40)	Curcumin + radiation Quasi-experiment	4 g/day, 7 days	↓survivin levels	(155)
**Endometrial cancer**				
Endometrial cancer		Inhibits proliferation of endometrial carcinoma cells by down-regulating ERK/c-Jun signaling pathway activity	↓ERK2 and JUN genes mRNA expression levels ↓inhibited phosphorylation of ERK and c-Jun	(156)
**Endometriosis (EM)**				
	A rats model of EM	Low, medium and high dose curcumin groups (60, 120, 240 mg/kg)	↓Local inflammatory ↓Ectopic endometrial invasion ↓Angiogenesis ↓Notch1 signaling pathway ↓Expression of MMP-9 and VEGF mRNA	(157)
	A mouse model of EM	Curcumin sesame oil solution (300 mg/kg) once daily for 21 days	↓ Number of lesions ↓Peritoneal volume and degree of adhesions ↓Peritoneal fluid levels of IL-6, IL-1β, and VEGFA ↓HIF-1α ↓VEGFA protein ↓VEGFA protein and gene levels	(158)
**PCOS**				
Women with PCOS aged from 18 to 40 years old (*n* = 60)	A randomized, double-blind, placebo-controlled trial	500 mg/day curcumin Placebo 2 weeks	↓Weight and BMI ↓Fasting glucose ↓Serum insulin ↓ HOMA-IR (insulin resistance), ↓Total cholesterol ↓ LDL-C ↓total cholesterol/HDL cholesterol ↑HDL-C ↑QUICKI (insulin sensitivity) up-regulated gene expression of PPAR-γ up-regulated gene expression of LDLR	(159)
Women with PCOS aged from 18 to 49 years old (*n* = 67)	A randomized, double-blind, placebo-controlled clinical trial	500 mg curcumin powder in a capsule Placebo (maltodextrin) capsules orally 3 times daily for 12 weeks	↓FPG ↓dehydroepiandrosterone levels Statistically non-significant increase in estradiol levels No changes in fasting insulin, LH and FSH, HOMA-IR	(160)
Women with PCOS		500 mg–1500 mg per day 6–12 weeks	↓NF-κB ↓TNF-α ↓IL-6 ↓DHEA ↑GPx ↓insulin	(161)
Prepuberal BALB/c female mice	DHEA was given to induce PCOS	5.4 mg/100 g curcumin, curcumin-loaded super-paramagnetic Fe3O4 nanoparticles, administered intraperitoneally for 20 days	↓ Oocyte volume ↓ Total primary, secondary ↓Central and primordial follicle number Moderate apoptosis of granulosa cells ↓BAX ↓CASP3 ↑Bcl2 ↓insulin	(121)
Wistar rats	Stradiol valerate administered	100, 200, 300 and 400 mg/kg 14 days	↑FSH ↑Progesterone ↓LH ↓Estradiol ↓Testosterone ↓IL-6 ↓TNF-α ↓CRP	(162)
**Premenstrual syndrome (PMS)**				
Women PMS and dysmenorrhea (*n* = 62)	A randomized, triple-blinded, placebo-controlled clinical trial	500 mg curcuminoid, one capsule, 500 mg daily from 7 days before to 3 days after menstruation for three consecutive menstrual cycles	↓Dysmenorrhea pain ↓ PSST	(163)
Women PMS and dysmenorrhea (*n* = 57)	Placebo-controlled clinical trial	One capsule containing 500 mg curcumin plus piperine was given daily until 7 days before menstruation for three consecutive menstrual cycles	↓Insomnia and sleepiness scores in both curcumin	(164)
Adolescent girls with premenstrual syndrome (PMS) and dysmenorrhea (*n* = 80)	Triple-blind, randomized, placebo-controlled study	for 3 consecutive menstrual cycles, from 7 days before to 3 days after the onset of menstruation, curcuminoids (500 mg + 5 mg piperine) were randomly administered daily	↑ Free radical scavenging activity of serum ↑Free radical scavenging activity ↑ Antioxidant potential	(165)
**Menopause**				
Healthy postmenopausal women (*n* = 84)	Three blind parallel randomized controlled trials 40–60 years	The curcumin group was given one capsule containing 500 mg curcumin twice daily for 8 weeks	↓Total menopausal symptoms ↓Depression ↓Anxiety ↓Psychological ↓Vasomotor score ↑Serum TAC levels ↓Serum MDA and hs-CRP levels	(166)
Healthy postmenopausal women with BMI 25–40	Randomized crossover study 125 g bioactive or placebo yogurt + curcumin was consumed	Blood was drawn at baseline, 30 min and 1, 2, 3, and 4 h after the meal	↓Plasma TNFα Cmax ↓Plasma TNFα	(167)

HOMA-IR, homeostatic model assessment of insulin resistance; QUICKI, quantitative insulin sensitivity check index; LDL cholesterol, low-density lipoprotein cholesterol; HDL cholesterol, high-density lipoprotein cholesterol; LDLR, low-density lipoprotein receptor; FPG, fasting plasma glucose; HOMA-IR, insulin resistance; hs-CRP, high-sensitivity C-reactive protein; HIF-1α, Hypoxia-inducible factor 1-alpha; TNF-α, Tumor Necrosis Factor Alpha; TAC, serum total antioxidant capacity; BAX, Bcl-2-associated X protein; CASP3, caspase3; Bcl2, B-cell lymphoma 2; MDA, malondialdehyde; TBARS, thiobarbituric acid reactive substances; GSH, glutathione peroxidase; SOD, superoxide dismutase; CAT, catalase; TNF-α, tumor necrosis factor alpha; PI3K, phosphoinositide 3-kinase; AKT, protein kinase B; mTOR, mammalian target of rapamycin; IL-6, interleukin-6; CRP, C-reactive protein; HbA1c, hemoglobin A1C; FSH, follicle-stimulating hormone; PPAR-γ, peroxisome proliferator-activated receptor-gamma; hs-CRP, high-sensitivity C-reactive protein; SOD, superoxide dismutase; VEGFA—Vascular endothelial growth factor A; BMI, Body mass ındex; PSST, Premenstrual Syndrome Screening Tool.

This review aims to critically evaluate curcumin’s therapeutic potential as an adjunctive agent in gynecologic oncology and benign gynecologic disorders, while also highlighting current limitations and future directions for clinical application.

## Methods

A comprehensive literature review was conducted on the health effects of gynecology. Relevant articles were identified through systematic searches in major biomedical databases, including PubMed/MEDLINE, Scopus, Cochrane Library, EMBASE, and WOS databases. The search strategy incorporated a combination of MeSH and free-text terms, including “polyphenol,” “curcumin,” “gynecological diseases,” “curcumin mechanisms of action,” “gynecology,” “ovarian cancer,” “cervical cancer,” “endometrial cancers,” “endometriosis,” “polycystic ovarian syndrome,” “premenstrual syndrome,” “menopause,” “antioxidant properties,” “anti-inflammatory mechanisms,” “anticancer mechanisms,” “antimicrobial effects,” and “hormonal and metabolic effects.”

Only full-text articles published in English were included in the analysis. After screening titles and abstracts, publications lacking sufficient relevance to the topic, publications in pre-print format, and publications in languages other than English were excluded. The selected studies were categorized as original research, review articles, systematic reviews and meta-analyses, case-control studies, or clinical studies. Human studies, animal models (*in vitro* and *in vivo*), and cell-line experiments examining the health effects of curcumin were thoroughly evaluated, including systematic, narrative, and meta-analytical analyses. This evaluation was conducted using a holistic approach to establish a scientific basis for the review.

## Curcumin: mechanisms of action

**FIGURE 1:**
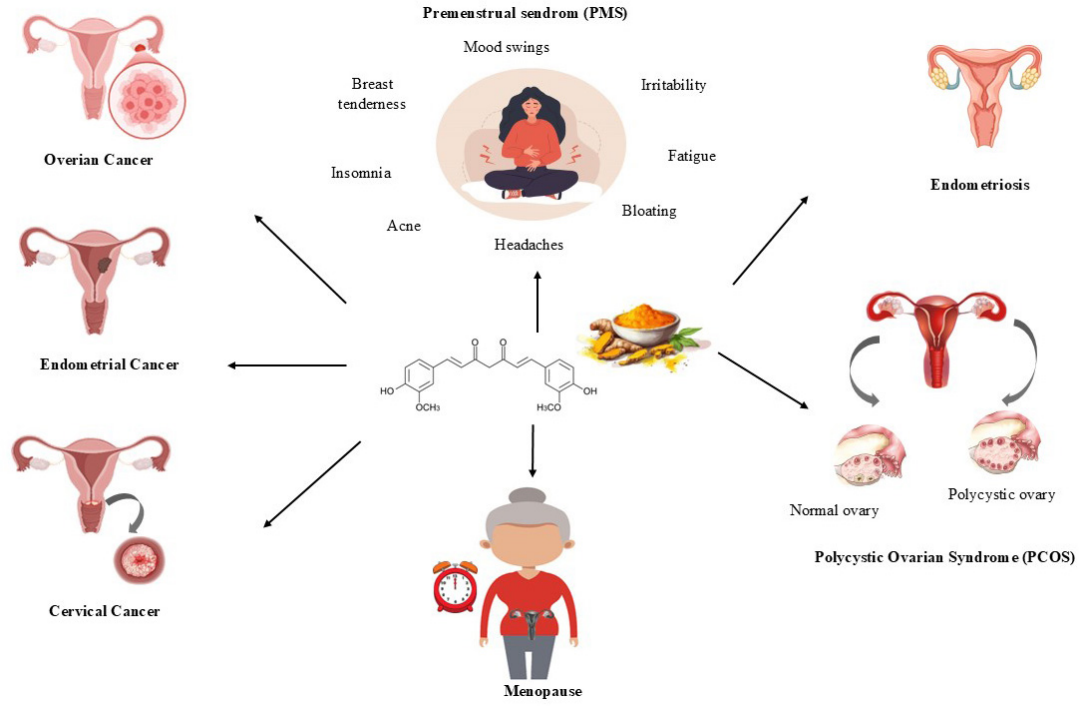
Curcumin-related gynecological diseases.

Curcumin, the main active ingredient in turmeric, has attracted interest due to its wide range of therapeutic benefits that extend beyond its recognized anticancer properties. This natural substance demonstrates anti-inflammatory, antioxidant, antimicrobial, and anti-atherosclerotic effects, positioning it as a promising option for addressing various gynecological disorders (1–9). The multifaceted mechanisms of action exhibited by curcumin highlight its potential to improve women’s health. An overview of these mechanisms is illustrated in Figure 1.

### Anti-inflammatory mechanisms

Curcumin demonstrates its anti-inflammatory properties by engaging with important receptors and altering various inflammatory signaling pathways. It attaches to TLRs, which are essential for the immune response, and affects downstream pathways such as NF-κB, MAPK, and AP-1 (15, 16). By blocking NF-κB, curcumin can significantly reduce the production of several pro-inflammatory cytokines (16). Additionally, curcumin interacts with PPARγ, which further diminishes NF-κB activity (17). Another key mechanism is the inhibition of the NLRP3 inflammasome, a multi-protein complex associated with inflammatory diseases (18). Curcumin can prevent the formation and activation of the NLRP3 inflammasome either directly or by inhibiting NF-κB signaling (19). Curcumin has been shown to significantly reduce the levels of various pro-inflammatory mediators in both experimental models and clinical settings. It decreases the production of cytokines such as IL-1, IL-6, IL-8, IL-17, and TNF-α (20–23). Inflammatory conditions are often exacerbated by dysregulated non-coding RNA networks and immune cell polarization, as seen in chronic diseases where exosomal lncRNAs such as MEG3 can drive M1 macrophage polarization and pyroptosis through pathways involving TREM-1 (24). Similar immunomodulatory pathways are also targeted by other traditional herbal agents, such as Ephedra sinica, which has been shown to regulate inflammatory and immune responses in conditions like wind-chill cold (25). Clinical trials support these findings (7, 8). A randomized, double-blind, placebo-controlled study demonstrated that daily supplementation with 80 mg of curcumin nano-micelles resulted in a statistically significant reduction in plasma levels of CRP and TNF-α (26). Curcumin’s ability to lower these inflammatory markers underscores its potential as a therapeutic agent for various inflammatory diseases (27, 28).

### Antioxidant properties

Oxidative stress, resulting from the buildup of ROS, plays a role in triggering and sustaining inflammatory responses. An excess of ROS can activate various transcription factors linked to inflammatory responses, exacerbating inflammation. Curcumin helps reduce oxidative stress by lowering ROS production through its effect on NADPH oxidase and boosting the activity of antioxidant enzymes (29). By lowering ROS levels and improving antioxidant capacity, curcumin supports its anti-inflammatory properties and promotes overall cellular health.

### Anticancer mechanisms

Curcumin exerts significant anticancer effects through modulation of critical signaling pathways implicated in tumorigenesis, particularly the NF-κB and signal transducer and STAT3 pathways. Additionally, curcumin may influence ion channel activity, such as TRPM2, which is implicated in tumor progression and represents a promising therapeutic target in oncology (30). Likewise, constitutive activation of STAT3, frequently observed across various malignancies, promotes tumor growth, invasion, angiogenesis, and immune evasion, further contributing to therapeutic resistance (31). Curcumin has been demonstrated to inhibit the activation of both NF-κB and STAT3, thereby disrupting key processes in cancer development and progression. In addition, curcumin suppresses colony formation in colorectal cancer cells, partially through the downregulation of Sp1, a transcription factor known to facilitate cancer cell proliferation (32).

### Hormonal and metabolic effects

Curcumin modulates several endocrine and metabolic pathways, notably those involved in insulin signaling and sex hormone regulation. It enhances insulin sensitivity by upregulating insulin receptor substrates and activating the PI3K/Akt signaling cascade, which is critical for maintaining glucose homeostasis and preventing insulin resistance (33). Through its potent anti-inflammatory activity, curcumin suppresses pro-inflammatory cytokines such as TNF-α, IL-6, and IL-1β, thereby mitigating the inflammation-induced disruption of endocrine function (34, 35).

Conversely, curcumin promotes the transformation of white adipocytes into a brown fat phenotype, thereby enhancing energy metabolism (36). It also interferes with steroidogenesis by inhibiting key enzymes such as 17β-hydroxysteroid dehydrogenase and aromatase, offering therapeutic potential in hormone-related conditions, including PCOS (37–39). Moreover, curcumin modulates neuroendocrine regulation by influencing hypothalamic and pituitary hormone release, with downstream effects on cortisol and leptin levels, which are pivotal for energy balance and the stress response (7, 40).

## Curcumin’s role in gynecological conditions

Curcumin has garnered considerable attention as a potential therapeutic agent in gynecology. Its pleiotropic pharmacological properties particularly anti-inflammatory, antioxidant, and hormonal regulatory effects render it beneficial in the management of various gynecological disorders, including PCOS, PMS, dysmenorrhea, and gynecologic malignancies.

### Gynecological cancers

Gynecologic cancers refer to various forms of abnormal cell growth within the female reproductive system, including endometrial, ovarian, cervical, primary peritoneal, vulvar, and vaginal cancers. It was reported that over 1 million new cases were diagnosed, leading to more than 580,000 deaths from endometrial, cervical, and ovarian cancers (41). While endometrial and cervical cancers are frequently diagnosed at earlier stages due to available screening and symptom presentation, ovarian cancer is often detected at an advanced stage, posing significant therapeutic challenges (13, 42). Despite progress in cancer treatments and the development of new therapies, including monoclonal antibodies used in immunotherapy, these cancers still present high mortality rates (43, 44).

### Effects of curcumin on ovarian cancer

Ovarian cancer includes a diverse range of tumors that vary based on their cell or site of origin, pathological grade, associated risk factors, prognosis, and treatment options (45–47). Conventional chemotherapy represents the primary therapeutic strategy for the management of ovarian cancer; however, its efficacy is constrained by significant adverse effects, temporary responses, and a high incidence of recurrence (48).

Notably, curcumin analogs such as DAPs have been synthetically modified to improve pharmacokinetics and biological efficacy. These analogs such as H-4073, HO-3867, H-4318, and HO-4200 demonstrate enhanced antiproliferative activity and greater pro-apoptotic potential compared to native curcumin in various malignancies, particularly ovarian and colorectal cancers (49–53). Mechanistically, curcumin exerts its anticancer effects in ovarian cancer through multiple pathways. It suppresses the activation of NF-κB and STAT3 while concurrently activating the Nrf2/heme HO-1 axis, thereby reducing tumor burden and enhancing cellular apoptosis (54).

Moreover, curcumin downregulates key signaling cascades involved in tumor proliferation, metastasis, and angiogenesis, including PI3K/Akt, Wnt/β-catenin, JAK/STAT3, and MEK/ERK1/2 pathways (48). In studies have further demonstrated that curcumin induces both apoptosis and autophagy in human ovarian cancer cell lines such as A2780 and SK-OV-3, and this dual activity is amplified when autophagy is pharmacologically inhibited (55, 56). Shakeri et al. confirmed curcumin’s antiangiogenic effects in animal models (57), while other findings have highlighted its role in modulating non-coding RNA networks particularly the circ-PLEKHM3/miR-320a/SMG1 axis to suppress tumor growth (58). Curcumin also interferes with EGFR signaling, thereby reducing Aquaporin 3 expression and inhibiting cell migration through downregulation of the EGFR/AKT/ERK cascade (59).

**FIGURE 2:**
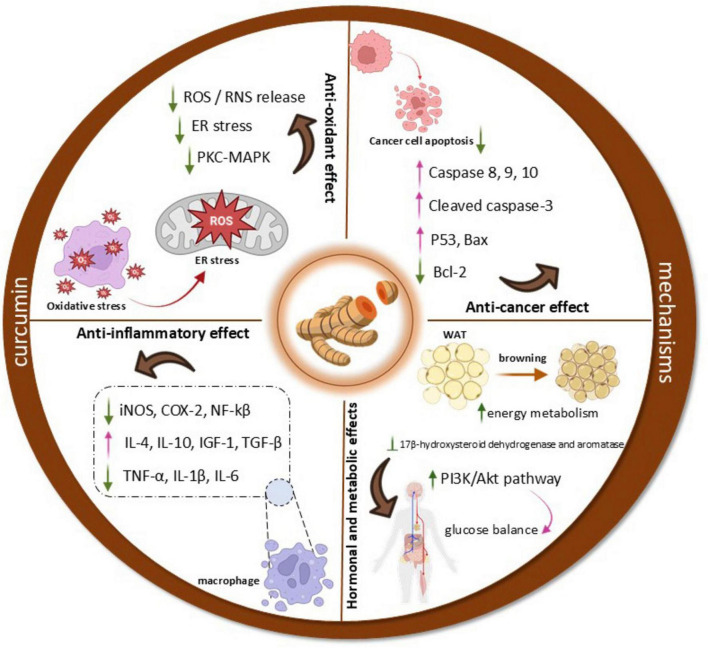
Schematic representation of curcumin’s proposed molecular mechanisms.

In addition, it enhances TRAIL induced apoptosis through the activation of both intrinsic and extrinsic pathways, potentially overcoming resistance to standard chemotherapy (60). Of particular relevance is curcumin’s effect on EOC spheroids, which are known to mediate chemoresistance and facilitate peritoneal metastasis (61). Curcumin significantly downregulates ALDH1A1, a marker of cancer stemness, thereby increasing cisplatin sensitivity, inhibiting spheroid formation, reducing invasion through mesothelial monolayers, and suppressing extracellular matrix adhesion (62). A schematic representation of the molecular mechanisms of curcumin is shown in Figure 2.

### Effects of curcumin on cervical cancer

Early-stage cervical cancer generally carries a favorable prognosis with high response rates to conventional therapies such as chemoradiation and surgery (63). However, these modalities can adversely affect ovarian and vaginal functions, underscoring the need for adjunctive or alternative treatments (64). Curcumin has demonstrated time- and dose-dependent cytotoxicity against cervical cancer cells, particularly those infected with high-risk HPV subtypes (65). Curcumin exerts its anticancer effects by targeting multiple signaling cascades, including the mitochondrial apoptosis pathway, inducible iNOS, COX-2, cyclin D1, ERK, Ras, and telomerase activity (66, 67). Evidence from studies indicates that curcumin exerts its anti-tumor effects through the modulation of various biological signaling pathways, including PI3K/Akt, JAK/STAT, MAPK, Wnt/β-catenin, p53, NF-κB, as well as apoptosis-related pathways. In addition, curcumin has been reported to inhibit tumor growth, angiogenesis, epithelial-mesenchymal transition (EMT), invasion, and metastasis by modulating the expression of tumor-associated non-coding RNAs (ncRNAs), highlighting its potential as a multi-targeted anticancer agent (68, 69). He et al. reported that the combination of curcumin with PDT and DAPT, a Notch pathway inhibitor, significantly enhanced apoptosis in Me180 cervical cancer cells. The observed effect was attributed to downregulation of NF-κB and Notch-1 pathways (70). Similarly, curcumin was found to suppress proliferation and invasion by disrupting the Wnt/β-catenin and NF-κB pathways in cervical cancer cell lines (71). At a concentration of 13 μM, curcumin induced DNA damage and chromatin condensation, leading to apoptotic cell death in HeLa cells (72). Synergistic approaches have further improved curcumin’s efficacy. For instance, combining curcumin with ultrasound significantly enhanced apoptotic activity in SiHa and HeLa cells, compared to curcumin monotherapy (73). Curcumin has also been shown to elevate intracellular ROS levels specifically in cervical cancer cells, triggering ER stress and promoting apoptosis, while sparing normal epithelial cells (74).

Moreover, curcumin and doxorubicin have both demonstrated dose-dependent inhibition of HeLa cell proliferation and induction of apoptosis over a 72-h period. This effect was accompanied by downregulation of RAF and RAS mRNA and protein expression, suggesting a potential combinatorial therapeutic strategy (75). Taken together, current evidence supports curcumin as a multifaceted therapeutic agent in cervical cancer management. Its mechanisms include the induction of apoptosis, inhibition of HPV activity, suppression of tumor proliferation and metastasis, and promotion of autophagy in malignant cells (76).

### Effects of curcumin on endometrial cancer

The development of EC is driven by a complex interplay of risk factors, including obesity, diabetes, family history, hormonal imbalances, PCOS, hormone therapy, use of intrauterine devices, and endometrial hyperplasia (77). In parallel with the growing interest in integrative cancer therapies, phytochemicals such as curcumin have been extensively explored for their anticancer properties (78, 79). Curcumin exerts potent anticancer effects in EC through multiple mechanisms. Curcumin has been shown to reduce cell viability, inhibit migration, induce apoptosis, and cause S-phase cell cycle arrest in Ishikawa cells. It downregulates mRNA expression of ERK2 and JUN, inhibits phosphorylation of ERK and c-Jun, and disrupts the ERK/c-Jun pathway, thereby suppressing endometrial carcinoma cell proliferation (80). In addition, curcumin decreases the expression of MMP-2, impeding extracellular matrix degradation and cellular invasion, while simultaneously upregulating E-cadherin, a key molecule in maintaining epithelial integrity (81).

Curcumin also targets anti-apoptotic molecules such as TREK-1 to reduce cell proliferation in EC cells (82). In *in vivo* models, including NOD-SCID mice, daily intraperitoneal curcumin injections (50 mg/kg) for 30 days led to a fivefold reduction in tumor volume without observed toxicity (83). Curcumin was also reported to inhibit EC cell migration by increasing Slit2 expression, which in turn downregulated pro-metastatic proteins like CXCR4, SDF-1, and MMP-2/9 (83). These actions are closely linked to the inhibition of ERK signaling, a pathway known to regulate cell survival, proliferation, and metastasis (84, 85). Curcumin significantly up-regulated the expression of Slit-2 in Ishikawa, Hec-1B and primary endometrial cancer cells, while it down-regulated the expression of SDF-1 and CXCR4, which in turn, suppressed the expression of MMP 2 and 9, thus attenuating the migration of endometrial cancer cells. Curcumin markedly increased Slit-2 expression in Ishikawa, Hec-1B, and primary endometrial cancer cells, while concurrently decreasing the expression of SDF-1 and CXCR4. This modulation subsequently suppressed MMP-2 and MMP-9 levels, thereby attenuating the migratory capacity of endometrial cancer cells (83, 86). Furthermore, both CUR-M and native curcumin reduce proangiogenic cytokines IL-6 and TNF-α while upregulating IL-10, contributing to an anti-tumor microenvironment (86, 87). Liposomal curcumin further inhibits NF-κB signaling in EC cells and reduces levels of caspase-3 and MMP-9 (88–90). In a zebrafish embryo tumor model, LC significantly reduced tumor progression by blocking NF-κB activity and its downstream inflammatory and pro-survival targets (88).

Curcumin also interferes with AR signaling, which plays a role in EC pathophysiology. In RL-952 cells, curcumin suppressed AR expression via modulation of the Wnt signaling pathway, thereby reducing cell proliferation and inducing apoptosis (91, 92). When used in combination with the aromatase inhibitor letrozole, curcumin synergistically enhanced apoptosis and suppressed Bcl-2 expression in *in vivo* EC models, indicating its potential as a chemosensitizing agent (93, 94). Compared to conventional chemotherapeutic agents, curcumin has demonstrated a favorable safety profile with minimal toxicity in both preclinical and clinical settings, positioning it as a promising adjunct or alternative therapy for endometrial cancer (95). Notably, in women with endometriosis, curcumin at concentrations of 30–50 μmol/L significantly reduced the growth and proliferation of estradiol-dependent endometrial stromal cells, suggesting broader reproductive applications (96).

## Curcumin’s impact on common gynecological conditions

### Effects of curcumin on endometriosis

Endometriosis is a chronic, inflammatory gynecological disorder characterized by ectopic endometrial tissue, often resulting in pelvic pain and infertility. Despite the availability of hormonal therapies, pharmacological agents, and surgical interventions, many patients experience suboptimal outcomes. As a result, plant-based compounds such as curcumin have garnered increasing attention for their therapeutic potential (97). Curcumin exerts anti-inflammatory effects primarily by inhibiting the NF-κB signaling pathway, which is typically upregulated in endometriotic lesions but minimally active in normal endometrial tissue (98–100). This inhibition reduces the expression of pro-inflammatory cytokines such as TNF-α, IL-6, COX-2, and TGF-β (101–104). Since COX-2 is associated with increased proliferation and resistance to apoptosis, its suppression by curcumin may contribute to reduced lesion growth (105–108). In a *in vitro* study, curcumin (10–50 μmol/L) significantly inhibited the proliferation of endometriotic stromal and epithelial cells, partly by lowering estradiol levels, a key driver of lesion progression (109).

Curcumin also modulates OS in endometriosis by reducing ROS, lipid peroxidation, and enhancing antioxidant defense via activation of the Nrf2-Keap1 pathway (110–112). Its antioxidant profile is comparable to that of vitamins C and E, reducing nitric oxide production and increasing glutathione and superoxide dismutase levels (113–115). Curcumin has demonstrated pro-apoptotic activity through upregulation of Bax and downregulation of Bcl-2, with subsequent activation of cytochrome c, caspase-9, and p53 (116). Dose-dependent reductions in lesion volume, weight, and vascularization have also been observed (94, 117).

### Effects of curcumin on polycystic ovarian syndrome

Polycystic ovary syndrome is a complex, multifactorial endocrine disorder and a primary contributor to anovulatory infertility, often accompanied by metabolic disturbances and elevated cardiovascular risk (118, 119). In animal models, curcumin reduces ovarian weight and volume, decreases the number and size of cystic follicles, and promotes follicular maturation and corpus luteum formation (120–125). Curcumin positively impacts metabolic parameters, which is critical given the high prevalence of insulin resistance and obesity in PCOS. Meta-analyses confirm that curcumin significantly lowers BMI and improves glucose metabolism by reducing fasting plasma glucose, insulin levels, HOMA-IR, total cholesterol, and CRP (35, 36, 96, 126). These metabolic effects are linked to curcumin’s ability to regulate pro-inflammatory cytokines IL-6, TNF-α, leptin, and adiponectinand enhance the browning of white adipose tissue, thereby improving energy metabolism (34, 36). Inflammation plays a central role in PCOS pathophysiology, particularly in the context of hyperandrogenism. Curcumin suppresses IL-6 and TNF-α levels and inhibits NF-κB signaling, contributing to improved insulin sensitivity and reduced androgen synthesis in granulosa and theca cells (127–129). Clinical studies further demonstrate reductions in CRP levels and improved inflammatory markers in curcumin-treated PCOS patients (130–133).

### Effects of curcumin on premenstrual syndrome and dysmenorrhea

Premenstrual syndrome and primary dysmenorrhea are prevalent gynecological conditions characterized by pain, mood disturbances, and systemic inflammation. Conventional pharmacologic treatments are often limited by side effects and variable efficacy, prompting interest in plant-derived bioactive compounds such as curcumin. Curcumin has demonstrated anti-inflammatory and antioxidant properties that may alleviate symptoms associated with PMS and dysmenorrhea. In randomized controlled trials, curcumin supplementation was shown to significantly reduce serum hsCRP levels, an established biomarker of systemic inflammation, without disrupting iron homeostasis (134). Additionally, curcuminoid treatment led to a significant decrease in serum NOx metabolites indicating its role in modulating oxidative stress and vascular function in affected women (135).

The mechanism of action includes inhibition of COX-2 and prostaglandin synthesis, which are central to pain and inflammatory pathways. Moreover, curcumin reduces lipid peroxidation, stabilizes hormonal fluctuations, and exhibits analgesic properties. Notably, curcumin has been found to increase serotonin and dopamine levels, which may account for its antidepressant effects and benefit in managing mood-related symptoms associated with PMS (136). A recent meta-analysis confirmed that curcumin significantly decreases PMS severity scores and reduces the intensity and duration of dysmenorrhea (137). In addition, studies investigating immunologic parameters reported that curcumin, particularly when combined with piperine, may lower serum levels in women with PMS and dysmenorrhea, suggesting potential effects on allergic predispositions (138). However, these effects were not accompanied by significant changes in anti-inflammatory cytokines such as IL-10 or IL-12. A systematic review further indicated that curcumin supplementation improved vitamin D levels and reduced serum IgE, AST, and direct bilirubin, supporting its multi-systemic regulatory role (139). Furthermore, oral administration of curcumin at doses of 500–1000 mg/day has been reported to reduce menstrual pain by inhibiting uterine COX activity and mitigating prostaglandin-mediated uterine contractions (140). Despite these promising findings, further high-quality clinical trials are warranted to establish the optimal dosage, duration of therapy, and long-term safety profile of curcumin in the management of PMS and dysmenorrhea (137, 138).

### Effects of curcumin on menopause

Menopause is characterized by a reduction in estrogen levels, resulting in elevated oxidative stress, systemic inflammation, and the emergence of various vasomotor and psychological symptoms. Due to its antioxidant and anti-inflammatory properties, curcumin has been suggested as a potential non-hormonal strategy for alleviating menopausal symptoms (141). The curcumin group showed significant reductions in serum MDA and hs-CRP, while TAC increased in both the curcumin and vitamin E groups.

These results indicate that curcumin may enhance oxidative and inflammatory status in menopausal women, whereas vitamin E demonstrated supplementary effects in reducing anxiety-related symptoms. Vasomotor symptoms, such as hot flashes and night sweats, represent some of the most prevalent and distressing features of menopause and have been linked to an elevated risk of cardiovascular disease (142, 143). A clinical trial by Akazawa and Maeda assessed the impact of curcumin and aerobic exercise on vascular endothelial function in postmenopausal women. Over 8 weeks, both curcumin supplementation (150 mg/day) and exercise interventions significantly improved endothelial function, highlighting curcumin’s potential cardioprotective effects in this population (144). Psychological symptoms such as anxiety and depression are also prevalent during menopause due to estrogen deficiency (145, 146). In a double-blind crossover trial conducted by Ghayour-Mobarhan, 1 g/day of curcumin administered for 30 days reduced anxiety scores in obese adults, though no significant effect was observed on depressive symptoms (147). Similarly, a study by Esmaily et al. reported reductions in anxiety and depression levels in postmenopausal women receiving curcumin, although the differences compared to placebo were not statistically significant.

Collectively, available evidence supports curcumin’s potential role in improving inflammatory and oxidative biomarkers, enhancing vascular health, and alleviating anxiety symptoms in postmenopausal women. However, further large-scale, well-controlled studies are needed to confirm its efficacy and to determine optimal dosing strategies.

## Daily Intake

Curcumin is classified as Generally Recognized As Safe by the United States Food and Drug Administration and is considered safe and well tolerated even when consumed up to 8 g/day (168). This contrasts with certain immunotherapies, such as nivolumab, which can lead to serious immune-related adverse events including cholangitis (169). Due to its positive effects on health, the daily adequate intake value of curcumin is 0–3 mg/kg/day, according to the Joint Expert Committee on Food Additives and European Food Safety Authority reports (170, 171). As a result, curcumin has been shown that intake of up to 12 g/day does not have a harmful effect on individuals. There is some concern about inhibition of certain enzymes involved in drug metabolism, potential DNA degradation, iron chelation, and the relationship between curcumin intake (172). Turmeric and curcumin are non-mutagenic and non-genotoxic. Oral use of turmeric and curcumin did not have reproductive toxicity in animals at certain doses. Studies on human did not show toxic effects, and curcumin was safe at the dose of 6 g/day orally for 4–7 weeks. However, some adverse effects such as gastrointestinal upsets may occur. Moreover, oral bioavailable formulations of curcumin were safe for human at the dose of 500 mg two times in a day for 30 days, but there are still few trials and more studies are needed specially on nanoformulations and it should be discussed in a separate article. In addition, curcumin is known as a generally recognized as safe substance (10).

## Challenges and limitations

Despite its promising therapeutic potential, curcumin exhibits poor aqueous solubility (∼11 ng/mL), chemical instability at neutral to alkaline pH, and low oral bioavailability due to rapid hepatic metabolism and systemic elimination. Innovative delivery systems, such as curcumin-loaded pickering emulsions, have been developed to enhance solubility, stability, and bioavailability (173–175). Following oral, intravenous, or intraperitoneal administration, curcumin is predominantly metabolized to glucuronide and sulfate conjugates in the liver and excreted via bile into the gastrointestinal tract (176, 177). Pharmacokinetic studies have shown that even with oral doses as high as 8,000–12,000 mg/day, curcumin remains undetectable in systemic circulation or is only present at nanomolar levels, while its metabolites appear in trace amounts (178–180). These results highlight the necessity for advanced delivery systems including nano-formulations, liposomes, phospholipid complexes, and micelles to improve the therapeutic effectiveness of curcumin. Furthermore, comprehensive investigations into the interactions between cellular structures and curcumin nanoparticles are required to elucidate the underlying mechanisms governing their cellular uptake. Biologically active compounds present in the diet may exert pharmacological effects that contribute to human health benefits. Further detailed studies, based on animal models and clinical trials, are required to enhance the efficacy, safety, and mode of action of curcumin in disease prevention and management (180).

## Future perspectives

The clinical translation of curcumin in gynecology requires a strategic approach that addresses its pharmacokinetic limitations while leveraging its broad biological activity. Future research should prioritize the development of advanced delivery systems, including nanoparticles, liposomes, and phospholipid complexes, to improve curcumin’s solubility, stability, and bioavailability. Such formulations may also enable targeted tissue delivery, thereby enhancing therapeutic efficacy and reducing systemic exposure. Given its multi-targeted anti-inflammatory, antioxidant, and anti-neoplastic properties, curcumin holds promise not only as a standalone phytotherapeutic agent but also as an adjuvant to conventional treatments. In gynecologic oncology, particularly in cervical and endometrial cancers, curcumin may be integrated into combination regimens with chemotherapy, radiotherapy, or hormonal therapies to potentiate treatment responses, mitigate therapy-induced inflammation, and potentially overcome drug resistance through modulation of key signaling pathways such as NF-κB, STAT3, and PI3K/Akt. Similar integrative strategies may be applicable in chronic inflammatory gynecologic conditions, where curcumin could contribute to long-term disease modulation with a favorable safety profile. To facilitate clinical implementation, robust translational research pipelines are needed. These should encompass well-designed *in vitro* and *in vivo* studies alongside large-scale, multicenter randomized controlled trials with extended follow-up periods, diverse patient populations, and standardized dosing and formulation strategies. Importantly, future clinical trials should explicitly evaluate curcumin within combination and adjuvant therapy frameworks to better define its clinical utility and positioning.

In parallel, mechanistic investigations should further elucidate the molecular interactions of curcumin and its metabolites, including drug–nutrient interactions, hormonal modulation, immune regulation, and effects on the tumor microenvironment. Integrating mechanistic insights with clinical outcomes may support the development of a mechanism-driven framework for curcumin-based interventions and guide patient stratification strategies. Collectively, these efforts may position curcumin as an effective, low-toxicity adjunct or alternative in the prevention and treatment of gynecologic diseases, while also establishing it as a prototype compound for the development of next-generation phytotherapeutics with enhanced translational and clinical relevance.

## Conclusion and recommendations

Curcumin demonstrates considerable promise as an adjunct in gynecologic care due to its anti-inflammatory, antioxidant, and antineoplastic properties. Preclinical and limited clinical data suggest potential benefits in the management of gynecologic malignancies, endometriosis, and PCOS, PMS. Its pleiotropic molecular mechanisms support its role in addressing complex pathophysiological processes. However, its clinical translation is hindered by poor bioavailability and a paucity of large-scale randomized controlled trials. Innovative formulations and delivery systems are needed to optimize systemic absorption and therapeutic efficacy. Although high-dose curcumin has been well-tolerated in human studies, further investigation is required to establish standardized dosing, safety profiles, and potential drug-nutrient interactions. Until such evidence is available, curcumin should be recommended primarily as a dietary component rather than a therapeutic agent. Future well-designed trials are essential to validate its role in gynecologic oncology and other reproductive health conditions.
